# Search Strategies in the Perceptual-Motor Workspace and the Acquisition of Coordination, Control, and Skill

**DOI:** 10.3389/fpsyg.2019.01874

**Published:** 2019-08-14

**Authors:** Matheus M. Pacheco, Charley W. Lafe, Karl M. Newell

**Affiliations:** ^1^Motor Behavior Laboratory (LACOM), School of Physical Education and Sport, University of São Paulo, São Paulo, Brazil; ^2^Motor Behavior Laboratory, Department of Kinesiology, University of Georgia, Athens, GA, United States

**Keywords:** motor learning, ecological psychology, dynamical systems, coordinative structures, intrinsic dynamics, individual-differences, exploration

## Abstract

In this paper we re-visit and elaborate-on the theoretical framework of learning as searching within the perceptual-motor workspace for a solution to the task. The central focus is the nature of search strategies to locate and create stable equilibrium regions in the perceptual-motor workspace and how these strategies relate to the emergent movement forms in the acquisition of coordination, control, and skill. In the ecological theory of perception and action, the enhanced stability of performance occurs through the attunement of the perceptual systems to the task dynamics together with modifications of action as task and intrinsic dynamics cooperate and/or compete. Thus, through practice in this search process, individuals adapt to the pick-up of task relevant perceptual variables and change their movement form according to the stability of the performed action and its outcome in relation to the task demands. Contemporary experimental findings have revealed features of the search process given the interaction of individual intrinsic dynamics in the context of task requirements and principles that drive the change – e.g., exploitation of more tolerant task-space solutions and emergence of compensatory mechanisms. Finally, we outline how the search strategy framework relates to traditional learning-related phenomena: including the dynamical pathways of learning, learning curves, factors of learning, individuality, motor development, and sport and rehabilitation interventions.

## Introduction

The study of motor skill acquisition is central to understanding the underlying processes of how behavior comes to be the way it is as well as to providing principles of the best approaches to intervene on it. Skill acquisition is, in our view, however, less an “acquisition” and more a “transformation” or “change” of the individual abilities to act in searching within the environment in pursuit of a task goal.^1^ This search process reflects and gives emphasis to the continually evolving dynamics of perceptual-motor processes in learning.

In this paper, we revisit and expand on the Search Strategies Approach to skill acquisition (SSA) (Newell et al., 1989). The general theoretical backdrop draws on the ecological theory of perception and action. The SSA provides a theoretical and operational framework to examine the time-dependent processes of change in motor and perceptual variables, both within and between trials, over a range of task categories.

### Traditional Assumptions in Skill Acquisition: Group- and Task-Based Learning

We can summarize the majority of early and current views on skill acquisition as theorizing on how movement capabilities of a group evolve given changes in the constraints of a task condition. Two assumptions are held in studying skill acquisition this way: that the summaries of the group represent changes of each individual and/or that the task conditions are sufficiently constraining for learning to be of the same kind for all individuals.

In this way, individuals homogeneously learn the skill, converging in behavior. It means that when comparing, for instance, constant and variable practice (Schmidt, 1975; Moxley, 1979; Braun et al., 2010), individuals within each group are more similar than between groups and that the process of change observed through the average behavior is representative of all individuals.

The assumption of within-individual processes being represented by between-individual measures disregards the fact that motor learning and development (skill acquisition) are non-ergodic processes (Molenaar, 2008). That is, skill acquisition is a non-stationary process and each individual does not necessarily have the same dynamics (e.g., Liu and Newell, 2015). Individuals differ in initial conditions (Zanone and Kelso, 1992, 1994; Kostrubiec et al., 2012) and demonstrate distinct patterns to the pathway of change in learning (Rop and Withagen, 2014; Pacheco et al., 2017; Pacheco and Newell, 2018b).

The assumption of convergence in behavior also disregards that the majority of tasks may be solved in a number of ways (i.e., redundant tasks, see Bernstein, 1967; Latash, 2012). For this reason, it is not necessary that individuals must converge to the same solution (learn the same pattern) even in similar task conditions (Pacheco and Newell, 2018c).

A framework on skill acquisition should avoid such group- and task-based assumptions in order to unravel the principles underlying the phenomenon. In considering learning as searching, one understands that differing initial conditions afford different perception–action relations leading to distinct pathways in learning. Also, similar initial conditions diverge through the discovery of informational variables and the link between these and actions. In both cases, redundancy in the task allows these different pathways to diverge.

### Skill Acquisition and the Ecological Theory of Perception and Action

**FIGURE 1:**
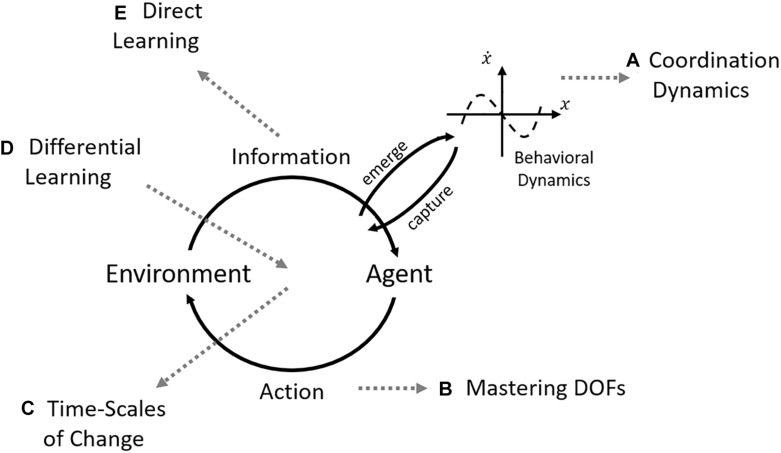
Focused efforts under DST on skill acquisition over the years. The central scheme is composed of the interaction between agent and environment through perception and action that is captured and gives rise to the coordination dynamics (adapted from Warren, 2006). The gray dashed arrows point to the different “themes” that emerged in investigating each aspect of the central scheme. **(A)** Coordination dynamics focused on the change of the intrinsic dynamics in practice (e.g., Zanone and Kelso, 1994; Kostrubiec et al., 2012); **(B)** there was a round of testing the freezing/freeing degrees of freedom (DOFs) hypothesis of Bernstein (1967) (e.g., Vereijken et al., 1992; Mitra et al., 1998; Newell and Vaillancourt, 2001); **(C)** formal analyses on the performance curves observed in motor learning (Newell et al., 2001, 2009); **(D)** the differential learning approach focused on concepts of random variability, stochastic resonance, and others to provide an intervention paradigm (Schöllhorn et al., 2009); and **(E)** the direct learning theory (Jacobs and Michaels, 2007) developed direct hypothesis on how perceptual learning would occur. Note that although all these approaches emerged from DST, they need to be integrated into a single framework (e.g., how does mastering the DOFs interact with performance curves studied in the time-scales of change?).

The ecological theory of perception and action offers a set of relevant constructs for motor skill acquisition. It provides a principled view on how the biological system interacts with its environment to achieve goal-directed behavior. Figure 1 shows the basic schematic (adapted) from Warren (2006) which illustrates the interaction between agent and environment through perception and action that gives rise to the emergent stable behavior at the coordination level. In incorporating the open non-linear nature of such interaction, the theory considers how intrinsic tendencies in perception and action of the individual change through interacting with the task requirements and environmental informational variables (Shaw and Alley, 1985). Additionally, it avoids the use of abstract internal processes to explain observed behavior as information for change (Fowler and Turvey, 1978) can be identified in the interaction between individual, task, and environment.

However, in our view, the current findings and propositions on perceptual-motor skill acquisition are not sufficiently integrated. The empirical work on different initial conditions of learners, for instance, is captured but only in a single paradigm (see Kostrubiec et al., 2012). Moreover, how different pathways emerge considering influences beyond the intrinsic dynamics has received limited attention. Another example is that perceptual learning – which has great influence on skill acquisition as a whole – has largely been considered in a separate literature (e.g., Withagen and Michaels, 2005; Bingham et al., 2014; but see Michaels et al., 2017). Figure 1 documents the different thematic directions of skill acquisition that the ecological theory has influenced.

The present paper builds-on the SSA to perceptual-motor learning. It sees skill acquisition as a search process in the perceptual-motor workspace (tendencies in perceiving and acting; intrinsic dynamics). The search process, then, induces modifications in these tendencies altering the movement capabilities of the individual; namely, skill acquisition. The SSA approach is within the more recent developments on ecological theory that already holds that the principles of acquisition cannot be derived from averaged results (dismissing between-individual variability, Withagen and Chemero, 2009) but in the commonalities that emerge from the differences between individuals. This view on acquisition encompasses divergent dynamics between individuals and holds the potential to unify the different foci presented in Figure 1. This paper presents current developments in search strategies together with how they relate to different foci of the contemporary motor learning literatures.

## Search Strategies Approach

The view of processes of change as search comes from an evolving broad perspective over many fields of study [see Hills et al., 2015 for a review]. We build here on the perspective of motor learning as the process of searching within the perceptual-motor workspace for a solution to the task requirements (Newell et al., 1989, 1991; Newell and McDonald, 1992; McDonald et al., 1995). The SSA was developed from the early ideas on complex systems by scholars of the former Soviet Union (Gel’fand and Tsetlin, 1962; Bernstein, 1967) and subsequently the evolving tenets of the ecological theory of perception and action (Fowler and Turvey, 1978; Gibson, 1979; Kugler et al., 1980; Turvey et al., 1981). The central focus of SSA is the means to locate and create stable and unstable equilibrium regions in the perceptual-motor workspace together with how these strategies relate to the emergent movement forms in the acquisition of coordination, control, and skill (Newell, 1985; Newell et al., 1989).

### Search

It is important to define what we consider as *search* behavior. Searching is characterized as systematic patterns between/within trials over time that emerge from the interaction between individual and environment in pursuit of the task goal. That is, patterns emerge from the search for solutions within the perceptual-motor workspace. Searching is the process of learning to attend to informational variables of the task (see Withagen and Michaels, 2005; Jacobs and Michaels, 2007) and modifying actions in terms of these informational variables resulting in changes of the perceptual-motor workspace.^2^ It should be noted that search occurs whether the individuals are practicing on their on (the practice is discovery-based) or “guided” or constrained by a coach, a teacher and so on (see the section “Learning Factors Through SSA”). The advance that we aim for is to identify how such systematic patterns emerge from the confluence of constraints.

Note that the approach relies on more than one level of analysis to characterize searching. This follows the long-held view point that there is not a one-to-one relation between movement patterns employed in a given task and the task outcome (Arutyunyan et al., 1969; Abbs et al., 1984; Kelso et al., 1984) and the fact that individuals actually exploit the many-to-one relation available (see Schöner, 1995). Thus, to understand searching in the perceptual-motor workspace, we consider more than the single level analysis of performance outcome. Procedurally, we explore a few key levels of description defined *a priori* in the attempt to identify informational variables (or contexts) that lead to given changes in action together with the actions that constrain perception of a set of informational variables.

An important feature in understanding search processes is the assumption that the observed search patterns are the entry to understanding the learning process. As individuals search through the space of possibilities, they attune to the most useful information and this modifies ways of acting that afford relevant information variables to be detected. Attunement refers to the process of attending to more useful information for the situation at hand^3^ while changes in action refer to changes in movement parameters to achieve the required response. This cycle of change on the many spaces, that we will define in the upcoming sections, is what modifies the capacity of perceiving and acting. In observing the emergence of these many systematic patterns in practice, we can understand a number of features. For instance, how individuals find or fail to find solutions; what are the informational variables that allow within-trial, trial-to-trial, and longer scales of change to occur; and how such systematic changes affect the perceptual-motor workspace.

On this process, we can understand what the aspects of the interaction between individual and task requirements are that lead to divergence during practice and learning. Nevertheless, we hold to the idea that individuals search in terms of attended information and action possibilities. Information is detected through interaction with the task and environment and action possibilities can be characterized. In capturing the commonalities in the individual emergent patterns of search, we seek to understand the general principles of learning.

### Spaces of Search: Goal, Task, and Perceptual-Motor Workspace

#### Perceptual-Motor Workspace

The characterization of *where*^4^ individuals search gives rise to investigation of the interaction between organism and task. Gibson (1966, 1979) proposed that the gradient and singular properties of the optic flow field arising from the individual–environment interactions provide the information for perceiving and acting. The theoretical view point of perceptual flow fields was taken to be generalizable to the other sensory systems. Kugler and Turvey (1987) were attracted to the idea that muscle-joint work spaces are organized by perceptual-motor fields that have similar invariant properties that provide information for perceptual-motor control.

In practice, it is postulated that individuals search through this *perceptual-motor workspace*. This is a characterization of the learner’s perception and action repertoire identified by stable and unstable possibilities of perception and action. These tendencies constrain, at least initially, how the search occurs, as performance in unstable regions is hard to maintain. The central hypothesis is that the information individuals use to search in the perceptual-motor work space is a macroscopic property defining the form of the layout of the gradient and singular regions emerging from both task-space and perceptual-motor workspace.

In the literature of ecological theory, different terms emerge to describe the tendencies in perceiving and acting. Here, we interchangeably use “intrinsic dynamics” and “perceptual-motor workspace.” The latter was coined to characterize the interaction between perceptual and action fields (Kugler and Turvey, 1987) while the former characterizes the emergent tendencies from these interactions (Kelso, 1995). Both, nevertheless, refer to “[…] relatively autonomous coordination tendencies that exist before learning something new” (Kelso, 1995, p. 163).

**FIGURE 2:**
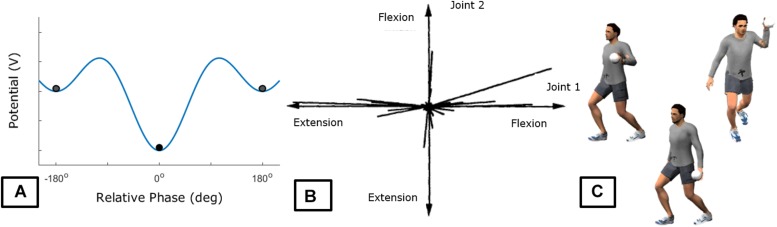
Exemplary intrinsic tendencies. Panel **(A)** shows the Haken et al. (1985) potential function that describes the tendencies observed for a majority of individuals in performing oscillatory motion of two limbs. As observed in **(A)**, the stable patterns that individuals can perform are the 0° (in-phase) or 180° (anti-phase). Panel **(B)** shows the distribution of covariation between two joints in the Krinskii and Shik (1964) paradigm utilized by Newell et al. (1991). There is a tendency to move in certain ways rather than using the whole possible space which is indicative of attractors within the space. This type of plot allows identification of tendencies in action when formal characterizations were not made but relevant variables are identified. Panel **(C)** is a schematic of different tendencies of action in terms of throwing patterns demonstrated in Pacheco and Newell (2018a). This description is qualitative given the difficulty to find relevant variables that characterize the different movement patterns (see Roberton and Halverson, 1984).

The most developed formal example in the literature of intrinsic dynamics is the Haken–Kelso–Bunz (HKB) model (Haken et al., 1985) that in describing a system performing oscillatory movements in two limbs demonstrated stability in two relative-phases: in-phase and anti-phase. This model implies that an individual will reliably perform the patterns of in-phase and anti-phase (for low frequencies) as they are stable feature of the intrinsic dynamics of the individual. The same does not hold for the 90° relative-phase which is not stable. For the 90° relative-phase be stabilized as a new attractor in the intrinsic dynamics, practice is necessary (Schöner et al., 1992; Zanone and Kelso, 1992). Note that individuals differ in terms of the intrinsic dynamics, leading to differences already when starting the practice (some individuals present stable performance of the 90°-phase pattern when starting practice, Kelso, 1995). Other approaches to describing intrinsic tendencies of the system to act are shown in Figures 2B,C.

**FIGURE 3:**
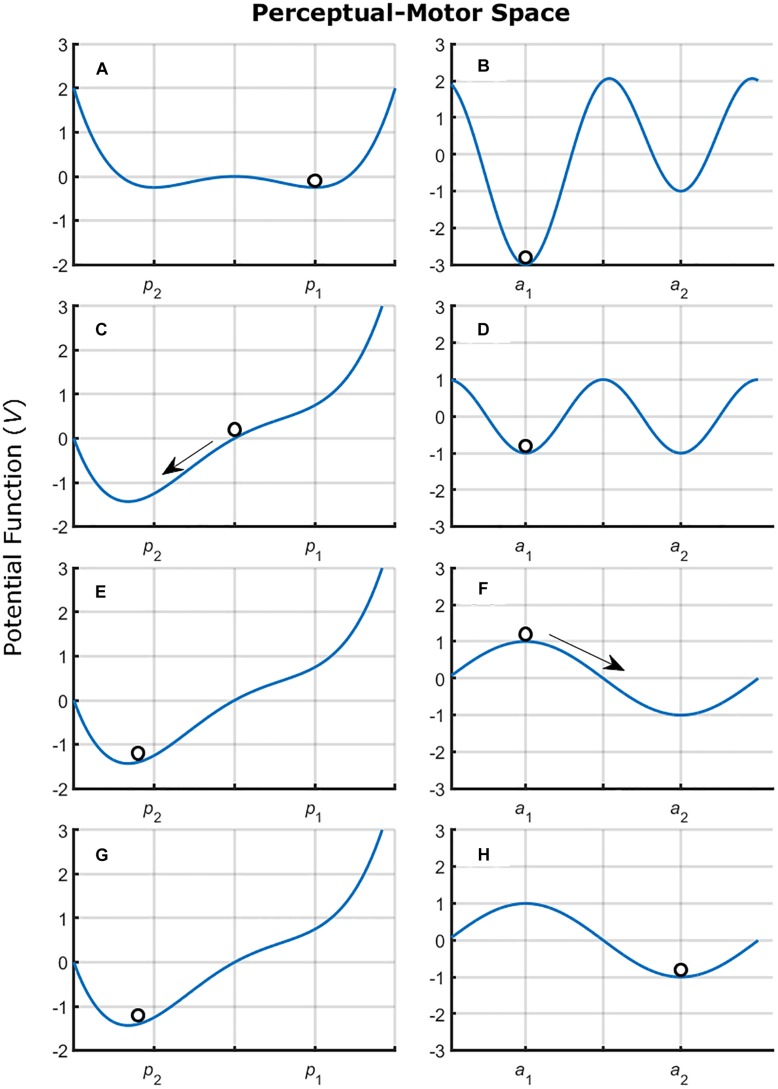
Graphical representation of a hypothetical perceptual-motor workspace. The potential function determines the stable states (valleys). The figure presents changes in the control parameter of the perceptual space **(A,C,E,G)** which results in changes in information attending from *p*_1_ to *p*_2_. The change in informational variable being attended alters the dynamics in the motor space **(B,D,F,H)** changing the stability of the action *a*_1_ and inducing changes to action *a*_2_. The perceptual space was characterized using Tuller et al. (1994) Eq. (1) *V*(*x*) = −*kx*−1/2*x*^2^−1/4*x*^4^ and the motor space was characterized using the Haken et al. (1985) Eq. (2) *V*(ϕ) = −*acos*⁡(ϕ)−*bcos*⁡(2ϕ). Both equations were coupled through variable *x* from Eq. (1), this alters Eq. (2) to *V*(ϕ) = −*xcos*⁡(ϕ)−(1 + *x*)*cos*⁡(2ϕ).

Note that all examples provided in Figure 2 describe “movement” variables and, thus, might be referring only to action tendencies (although see Schöner et al., 1992). There is a current discussion on whether there is a one-to-one relation between informational variables and perception (Withagen and Chemero, 2009) that can be extended to the relation between perception and action as well. Thus, although we are aware that the stable “movements” emerge from the perception-action coupling, the SSA assumes, in line with current evidence (e.g., Michaels et al., 2008; Withagen and van Wermeskerken, 2009; Rop and Withagen, 2014), that individuals can attend to more than a single informational variable. These informational variables vary on how useful they are for control of a given action. Thus, in order to *fully* describe the intrinsic dynamics, one must also consider the tendencies in *perception*. Furthermore, we need to determine whether the action tendencies are modified or not by changes in informational variable being attended. Figure 3 shows a hypothetical example.

The characterization of the perceptual-motor workspace is challenging. The HKB model holds given the robustness of the phenomenon and the simplicity of the experimental paradigm and model. There are only two degrees of freedom of interest and the model considers a single variable (relative-phase) that is constrained in the 0 to 2π range. Thus, assessing the intrinsic dynamics here appears straightforward: test all possibilities (see Zanone and Kelso, 1992). Nevertheless, if one wants to consider how individuals actually search through movement parameters to find a solution in the HKB paradigm, the task becomes rather complicated (see Michaels et al., 2017; Brakke and Pacheco, 2019). For movement patterns with larger number of degrees of freedom, it becomes almost impossible to even consider all possibilities in action to be tested. Figures 2B,C exemplify other strategies that have been used to characterize observed stable action patterns. The complexity of such description is further increased when one remembers that we are not, yet, considering the perceptual variables that can be attended to in such paradigm.

The intrinsic dynamics are task specific and only after a long round of research on the many constraints (biomechanical, physiological, neuronal) that decrease the space of possibilities will a full description be possible. Here, by analyzing how individuals approach a task, we provide a strategy to assess a limited region of the perceptual-motor workspace supporting performance of the task.

The perceptual motor workspace is ever evolving across the lifespan and at the same time is open to the influence of intention. The perceptual-motor workspace is non-stationary. There is a fundamental process via which practice modifies and, in turn is influenced by, the dynamics of the perceptual-motor workspace. Through practice and, more generally individual interactions with the environment, the perceptual-motor workspace is modified. That is, action continuously modifies qualitatively and quantitatively the landscape dynamics of the perceptual-motor workspace. The search through the perceptual motor work space changes through attunement to the information in the perceptual array and mapping of movement to it. In the context of action this mechanism of change channels the search strategy within and/or between trials of practice. This complementary process of the perceptual-motor workspace maps to the Gibson (1979) theoretical position that we perceive in order to move and that we move in order to perceive. The relation of search and change in the perceptual-motor workspace, however, is only beginning to be unraveled (see the section “Exploration of the Task-Space and Transfer”).

#### Task-Space and Goal-Space

Although individuals modify their bodily actions in terms of attended information and environmental constraints, there is a goal that also constrains the system into a coherent form. *How* the task goal constrains the behavior can be observed through a formalism called *task-space*.

Simply put, the task-space relates the movement possibilities and performance. Let us use the example of throwing a paper ball into a target on the floor. If we consider variation along a single dimension (e.g., the antero-posterior direction), the place where the ball lands determines the performance directly, *E* = *l*−*T*_p_ where *E* is the error, *l* is the landing position, and *T*_*p*_ is the target position.

**FIGURE 4:**
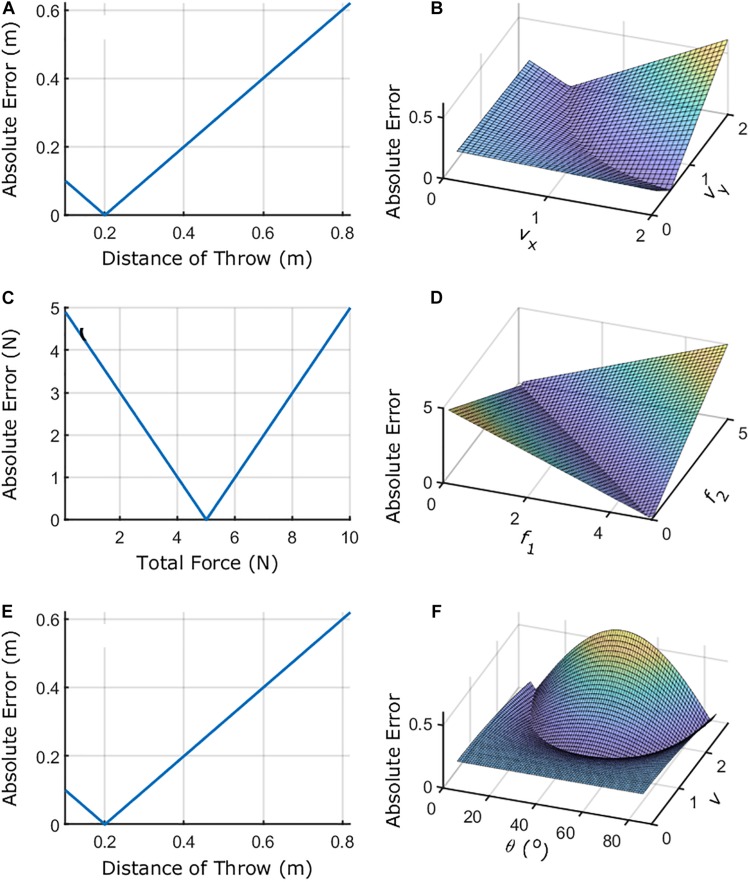
Schematic of task-spaces. Panels **(A,B)** show the outcome/performance and elemental variables (*v*_*x*_ and *v*_*y*_)/performance relations for the virtual throwing task where initial position is constant, and the target is located at 0.2 m from the initial position. *v*_*x*_ and *v*_*y*_ represent the component of the release velocity vector considering the *x-* and *y*-axes. Panels **(C,D)** show the outcome/performance and elemental variables (*f*_1_ and *f*_2_)/performance for the bimanual isometric force task when the total force target is 5 N. *f*_1_ and *f*_2_ represent the forces exerted by the index finger of each hand. Panels **(E,F)** show the outcome/performance and elemental variables (θ and *v*)/performance relations for the virtual throwing task where initial position is constant, and the target is located at 0.2 m from the initial position considering different coordinates. θ and *v* represent the release angle and speed. For the equations defining each task-space, see the text.

This representation of the task-space is overly simplistic if we want to understand how individuals alter their movements. We might get more insight into movement coordination by including variables of the movement that influence directly the outcome of the task. For instance, we know that the release velocity of the ball (assuming constant release position) determines the distance of throw (the landing position) – i.e., (2*v*_*x*_*v*_*y*_)/*g* = *l*, where *v*_*i*_ is the release velocity in the *i* axis (*x* – horizontal, *y* – vertical) and *g* is the gravity. We can make our preliminary task-space model more complex by introducing the release velocities *E* = (2*v*_*x*_*v*_*y*_)/*g*−*T*_p_. This is still simplistic as it only includes the release velocities of movement, but it provides clues as to how individuals might be organizing their movements. We could go on and try to make the model accommodate more dimensions, but this would not make it easy for visualization or analysis. Figure 4 shows how the task-space becomes more informative as we add complexity to the task-space for throwing and isometric force tasks.

We usually call *v*_*x*_ and *v*_*y*_ in throwing or *f*_1_ and *f*_2_ in bimanual isometric force (Figures 4A–D) as elemental variables. Latash et al. (2007) states that the choice of the elemental variables must conform to the fact that these are independently controllable.

Note that the task-space does not determine how an individual performs. Variability between individuals is quite common in terms of regions of the task-space where individuals perform and, again, is observed in the individuals’ interaction with the task. Also, as stated in the section “Traditional Assumptions in Skill Acquisition: Group- and Task-Based Learning,” although the task-space is a source of information for individual to achieve the goal, it does not determine how individuals act as, in some situations, individuals must move away from some valleys in the task-space (decrease performance/increase error) to find solutions more appropriate to its own intrinsic dynamics and/or improve performance further.

It could also be that there are transformations that better characterize the way individuals control their movement in a given task. For instance, the throwing paradigm can be characterized by *v*^2^*sin*⁡(2θ)/*g* = *l*, where *v* is the release speed of the ball and θ is the angle of release (Figures 4E,F). It could be that some individuals only modify *v* over time (maintaining θ constant) which would be simple to observe using these variables rather than *v*_*x*_ and *v*_*y*_ as in the other example.

Another important manifold to describe is the *goal-space*. This is the region described by zero error [e.g., *E* = 0 = (2*v*_*x*_*v*_*y*_)/*g*−*T*_p_]. From this, we can see that, when introducing the elemental variables to characterize the task-space, the goal can be achieved in infinitely many ways. That is, the goal-space is not described as a single point within the task-space. Many combinations of *v*_*x*_ and *v*_*y*_ allow the same zero-error outcome. This is also the case for the isometric force task presented in Figure 4D.

Tasks that have a goal-space with dimension higher than zero (i.e., they are not described by a point) are usually called *redundant* tasks (Sternad et al., 2014). This is because the goal can be achieved in many ways. In these tasks, an individual can exploit the degeneracy (i.e., structure-to-function ratio of many-to-one, Mason, 2010) that the biological system affords and demonstrate motor equivalency. Note, however, that not all tasks are redundant. These would be the tasks that do not include precision, but maximize aspects such as speed, force output, etc. For instance, if there is a maximum throwing distance a given individual can reach, given his/her physical abilities and biomechanical constraints, there is theoretically a unique movement pattern that will achieve it.

## Empirical Demonstrations of Ssa

In this section we illustrate how the SSA can characterize the perceptual-motor workspace and reveal important characteristics of search through reanalysis of previously published data. The first example comes from a simple application of the throwing study discussed earlier: a virtual throwing task performed on a computerized tablet where participant’s movement provides the release velocity of a ball. The goal of the task was to hit a target with the ball (Pacheco and Newell, 2015). The second example comes from an actual throwing task to a target to illustrate how the search can be studied with many-degrees-of-freedom tasks (Pacheco and Newell, 2018c). The third example comes from a re-examination of the within-trial dynamics from a single trial in a bimanual isometric force tracking task (Lafe et al., 2016). Note that, although we provide in each case a single-subject example, the same analyses can be (and were) used to identify differences between individuals.

### Virtual Throwing Task (Pacheco and Newell, 2015)

**FIGURE 5:**
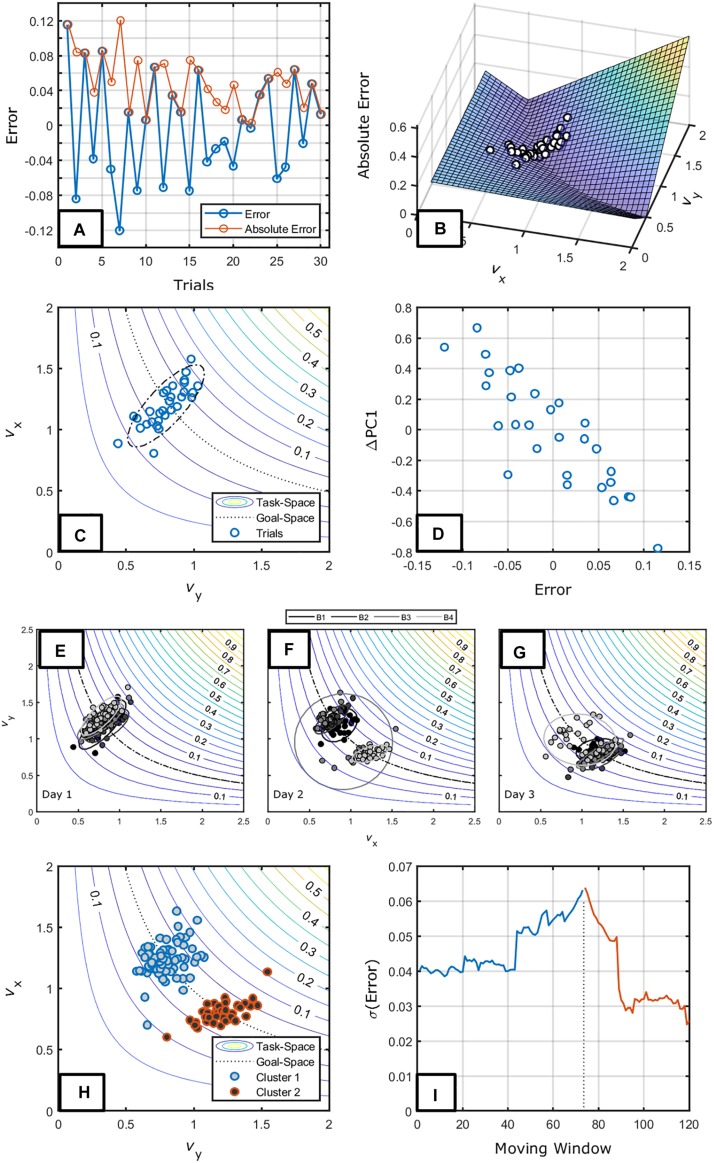
**(A)** The performance score of an exemplary participant in Pacheco and Newell (2015). The red line and circles represent the absolute error while the blue line and circles represent the errors. **(B)** The task-space plotting the same trials. **(C)** A contour plot showing the same trials and an ellipsoid highlighting the tendency to vary along a single dimension in the plane. **(D)** The relation between change along the main axis of variation of data and error. The data seem to indicate a proportional relation between change and error. Panels **(E–G)** show the long-term changes in distribution of the data in the task-space over blocks of 30 trials for days 1 **(E)**, 2 **(F)**, and 3 **(G)**. Panel **(H)** shows the identified two clusters of release velocities in the task-space for day 2. Panel **(I)** shows the moving window of standard deviation in performance before and after the change from cluster 1 to cluster 2. This shows the increased variation that might have induced changes in coordination pattern employed during the task.

Figure 5 shows exemplary data of the first 30 trials of a participant from Pacheco and Newell (2015). Figure 5A shows a decrement in error (absolute distance from target) and Figure 5B shows the same data in terms of the task-space using *v*_*x*_ and *v*_*y*_ as elemental variables. Given this plot, we can note a number of features.

The first is that this participant showed a “bracketing strategy” – a tendency to decrease error with alternating signs (Ellis and Wade, 1968; Liu et al., 1999). This can already differentiate different aspects of search since individuals can also demonstrate “creeping” (a decrease in error with the target being approached with constant sign), no change overtime or, even, get worse over time.

Second, this individual varied along a single dimension (even though he/she was free to explore the whole plane). We can infer that this dimension reflects the coordination that this individual is employing in this task. Performing a principal component analysis (PCA), we see that, indeed, this dimension represents 89% of the trial-to-trial variability (Figure 5C). A consequence of this analysis is that we can consider that the individual uses *mainly* this dimension to perform the task.

Considering the most prominent axis of variation we can investigate the relation between change and feedback. To do this, we see whether the *change* within the main axis of variation occurred as a function of the feedback received. Figure 5D shows the relation and this individual modified the scaling within the coordination mode proportionally to the feedback magnitude (*r* = −0.86, *p* < 0.001). A question that we might ask is whether the second dimension (orthogonal to the main axis of variance) is just inherent variability of the system or it has some role in this task. The former possibility is more plausible: it does not change in terms of performance (*r* = 0.10, *p* = 0.609) and its dynamics does not seem to show any structure (i.e., its autocorrelation shows a white noise pattern).

Does this individual change his/her coordination pattern over time? Figures 5E–G show the ellipsoids of data for each block of 30 trials (for the 3 days of practice) and make it clear that indeed the individual changed the region of the task-space as a function of practice. It is interesting to note that this was not a continuous change – there are no trials connecting the two data clouds. One ellipsoid for day 2 and one for day 3 (blocks 3 and 4, respectively) show a large area exactly because it includes both separate data clouds. We can then infer that the subject has two stable coordination patterns apart from each other and, during practice, he/she changed from one to the other. The two patterns are differentiated in Figure 5H.

What is the informational variable that induces changes between coordination patterns? More broadly, what is the context that makes individuals stop trying to correct the movement in terms of scaling the same coordination pattern to change coordination patterns? We anticipate that such information is a higher-order variable (variables derived from trial-to-trial KR, see Fowler and Turvey, 1978 and the section “Factors Modifying the Motion Through the Space”). In Pacheco et al. (2017), we found that increased variability predicted change in strategy. Here we see a steep increase in variability (standard deviation of performance) before coordination changes in day 2 (Figure 5I) and a fast decrease thereafter that could be indicative of a search for a more stable pattern.

### Throwing Task (Pacheco and Newell, 2018c)

We have been studying similar aspects in so-called whole-body movement tasks. Data from the experiment of Pacheco and Newell (2018c) allow such a method to be expanded to many degrees-of-freedom; it increases complexity in the analyses, but the results followed similar patterns.

**FIGURE 6:**
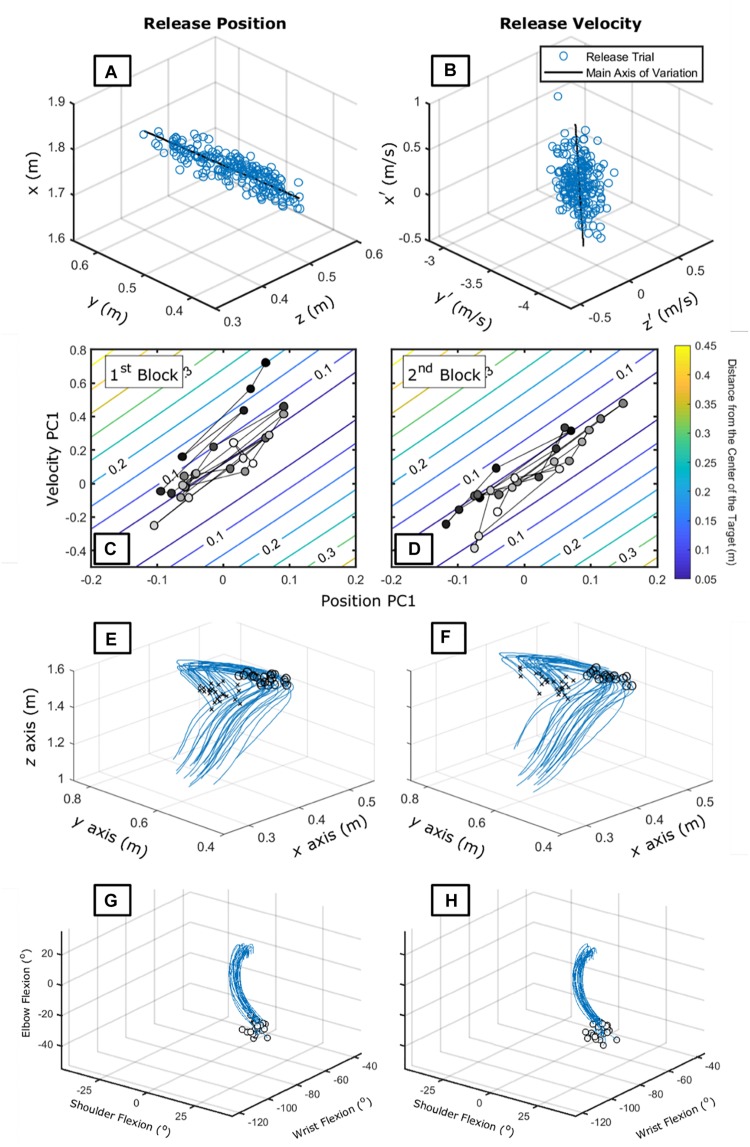
Panels **(A,B)** show the release parameters [position – **(A)** and velocity – **(B)**] of 210 trials of an exemplary participant in his 5th day of practice from Pacheco and Newell (2018c). The black line shows the main axis of variation. Panels **(C,D)** show the task-space plotted in terms of the first principal component of both velocity and position data for the first **(C)** and second **(D)** block of 25 trials. Darker circles represent earlier trials while lighter circles represent later trials. Panels **(E,F)** show the hand trajectory data for the first **(E)** and second **(F)** block of 25 trials. The *x*s represent the beginning of the recording and the circles represent the release position. Panels **(G,H)** show the joint motion relation between shoulder, elbow, and wrist for the first **(E)** and second **(G)** block of 25 trials. The circles represent the release position. For shoulder, 0 degrees mean the neutral position; for elbow, 180 degrees mean full extension; for wrist, 0 degrees mean neutral position.

If we want to characterize this task-space in terms of the release parameters – which would be the smallest set of elemental variables to use – we would not be able to plot it; it has six dimensions (a 3D velocity vector and 3D position vector). Thus, our approach is to find the coordination pattern between these variables before plotting it.^5^ Figures 6A,B show that this subject produced variation in a single dimension for both velocity and position – which is further confirmed by a PCA (92 and 72% of variance accounted for position and velocity data, respectively). Using these dimensions to build the task-space, as in Figures 6C,D, allows exploration of more features of the search process employed by this individual.

First, we could have decreased the dimension even more as this individual had, at least in the first two blocks of trials, a large correlation between velocity and position first PC. However, information on how the individual approached the goal-space (Figure 6C) would be lost.

Second, we see that this individual – different than others (Pacheco and Newell, 2018c) – managed to coordinate the release parameters along the goal-space direction. Such a pattern is clearly different than the one shown in the previous example (the tablet task). There, the main axis of variation was used to correct trial-to-trial deviations from the target (Figure 6D). Here, the *second* main axis of variation showed a slow convergence to the goal-space.

We can connect these different patterns to hypothesized processes of learning. Figure 5C reflects a process of *parameterization* within a coordination pattern – maintenance of the coordination pattern with a change in scaling (Newell, 1985). Figure 6C holds parallels to a process called *shift* (Kostrubiec et al., 2012) in which the whole coordination pattern is altered slightly to accommodate new task requirements. That is, the former characterizes change *within* while the latter a change *in* the main axes of variation. A *bifurcation*, where a new solution is stabilized over time, would be characterized by a broad distribution taking shape in the task-space around the transition (see Liu and Newell, 2015).

There is potential to investigate patterns that might be observed at other levels of description – such as the hand trajectory and joint motion. In Figures 6E–H there is not much change in the first two blocks of practice. Nevertheless, adaptations in terms of the whole trajectory of the hand in throwing (Cohen and Sternad, 2012; Nasu et al., 2014; Zhang et al., 2018) and joint motion to the task-space (Domkin et al., 2002, 2005) have been described. Little is known, however, how changes at that level emerge from ongoing perception of informational variables of the task-space.

### Bimanual Isometric Force Task (Lafe et al., 2016)

**FIGURE 7:**
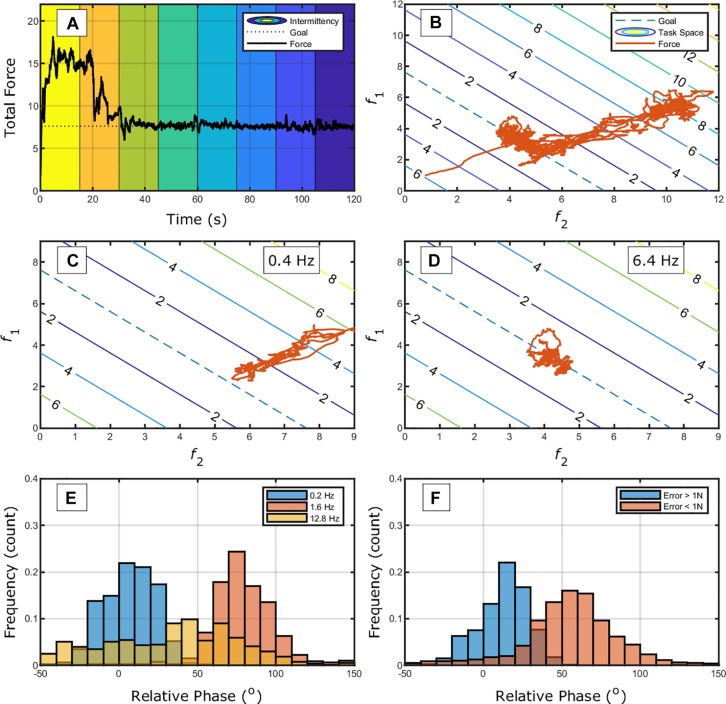
**(A)** Exemplary trial (first 120 s) of an individual performing the bimanual isometric force task with changing frequency of information (from left to right: 0.2, 0.4, 0.8, 1.6, 3.2, 6.4, 12.8, and 25.6 Hz, represented by different background colors) from Lafe et al. (2016). **(B)** The same trial now plotted in terms of the task-space. Panels **(C,D)** show the change in the elemental variables (*f*_1_ and *f*_2_) for different regimes of information intermittency; **(C,D)** for 0.8 and 1.6 Hz, respectively. Panels **(E,F)** show the relative-phase distributions for different information intermittency **(E)** and error magnitude in the task.

The SSA approach can also provide information in other time-scales of practice (e.g., within-trials). In Lafe et al. (2016), the task was to bimanually press two load cells each with an index finger to maintain a constant total force output while the frequency of concurrent feedback was manipulated. Figure 7A shows the data of total force over time through the different intermittent regimes of feedback. The figure shows that the individual had higher error for less frequent concurrent feedback.

Figure 7B shows the change in elemental variables in the task-space. It can be seen that there are two main axes of variation, one orthogonal and one along the goal-space. If we select two intermittency regimes (Figures 6C,D), two different coordination modes emerge. We can investigate whether these two types of coordination between hand emerge as a function of information intermittency. Figure 6E shows three intermittency regimes and a tendency for in-phase coordination for low frequency of information, 90°-phase for 1.6 Hz of information, and a more distributed relation for higher frequency. Thus, it could be that the frequency of information facilitates different coordination regimes.

Observing Figures 7C,D, one can raise the possibility that different coordination modes emerge, instead, as a function of error. That is, a clear in-phase pattern occurs to correct for errors and a more distributed pattern of phases occur around the target. Figure 7E differentiates phases according to the error magnitude. It shows that the error and coordination modes relation seems to hold as well. Clearly, as information is modified, the possibility to correct and act employing given coordination patterns is modified (Lee et al., 2019). A more fruitful investigation might be to manipulate the interaction between information and correction requirements.

### Insights From the Empirical Demonstrations

These examples show how the process can begin with identifying patterns in data to elucidate the interactions between individual and task requirements that give rise to the observed patterns. A further step would be to manipulate such aspects to confirm that our inferences hold. For now, these diagnostics show how individuals approached the task and we can infer about what were the intrinsic coordination tendencies and ways of interacting with information from task. This kind of information is of great importance to understand how individuals will or can change in a given practice situation. For instance, the individual in the section “Virtual Throwing Task (Pacheco and Newell, 2015)” demonstrated a coordination between the two elemental variables and searched along this single dimension following performance proportionally.

As discussed in the section “Throwing Task (Pacheco and Newell, 2018c),” the SSA can integrate processes of learning that are considered separately. We inferred about processes of learning such as *shifts*, *parameterization*, and *bifurcations*. Shifts in the landscape of the intrinsic dynamics have been analyzed in the realm of between-limb oscillation tasks (Kostrubiec et al., 2012) but have not been investigated in other tasks. Also, the SSA can integrate within a single approach the different forms of change in learning. Shifts and bifurcations refer to coordination pattern changes and parameterizations refer to learning when a given coordination pattern is already in the motor repertoire (Newell, 1985). We also demonstrated changes between already learned movement patterns used to perform the task (Figure 5H). Although this is *clearly* part of the process of learning in that individuals explore the action tendencies to find the most useful patterns to a situation, it is rarely discussed in the literature (but see Hristovski et al., 2011; Silva et al., 2019).

In the continuous force task example, the majority of analysis was at a time-scale much longer than the frame-by-frame time-scale of the task. This is because one must consider how corrections, fatigue, force variability, and processes of other time-scales interact to result in the observed pattern over time. The search analysis, however, showed that different coordination patterns (search patterns) emerge as the task allows (information availability) and requires (error magnitude) (see Lee et al., 2019). A practice study on the bimanual isometric force tracking design would provide how the relation between coordination patterns and task constraints emerge from searching, for instance, on a trial-to-trial basis. Clearly, an expansion must be to accommodate all these different time-scales encompassing the limits of perception and action loops to correct the movement (when the type of movement allows search within or only between trials – e.g., ballistic movements).

The SSA still seeks to identify the informational variables guiding the changes or the interactions giving rise to all the systematic patterns observed (e.g., jumps between movement patterns, shifts, etc.). However, even the consideration on limited data encompassing learning as searching provides interesting and fruitful directions for research.

## Investigating Skill Acquisition (Change) as Search

### Dynamical Principles of Change

Here, we evaluate potential principles of search in practice that result from our initial studies and those of others on the topic.

#### Perception and Action Search Within a Single Level

This first point is less a principle and more an assumption. Considering the many ways individuals can search to achieve a task goal we have been considering that individuals search in terms of a given “level” of analysis in a given time window (considering constant task constraints, see Hristovski et al., 2011). For instance, imagine that a soccer player is in a training situation where his team is attacking, and he/she possesses the ball. He/she can vary parameters within a given kicking movement pattern to score, change between kicking movement patterns, change in terms of the goal of its action such as passing the ball to others or kicking to the goal, etc. In our analysis, we have assumed that he/she might be searching only within a given level at a given time window and its search patterns will demonstrate that – as well it will demonstrate when search occurs in another level.

This assumption is in line with the view that individuals can pick up higher-order variables (informational patterns picked-up over time and space) emerging from the search in the task-space and perceptual-motor workspace. Through this process, information about changing at each level is detected. In explaining the requirement of information to guide motion through the task-space (see the section “Factors Modifying the Motion Through the Space”), Fowler and Turvey (1978) hypothesized that individuals would parameterize a given movement coordination in terms of change in performance (Δ*E*), would change coordination pattern in terms of the change of change in performance [Δ(Δ*E*) where *E* means error or the performance in the task]. Gel’fand and Tsetlin (1962) also proposed that Δ*E*/*E* would call for non-local change to avoid local-minima. Thus, individuals might be acting in terms of Δ*E* (within a coordination pattern) up to the moment that Δ*E*/*E* crosses a given threshold and an overall change in action is required (altering the level of search).

The dynamics of this change could follow the dynamics exemplified in Figure 3. That is, the interaction with the space could modify the tendency of an individual in using a given informational variable changing to another variable. In its turn, this would modify the tendency to act to another movement pattern. Although we are not sure about the exact dynamics of such changes, discontinuities in search have been recently observed (Pacheco et al., 2017). We found such discontinuities in terms of the section “Virtual Throwing Task (Pacheco and Newell, 2015)” where an individual, in realizing the inefficiency of a given movement pattern, “jumped” to another one that generated a decreased variability in performance.

A consequence of studying different levels of search is that we have the opportunity to investigate the exploration/exploitation dilemma – the dilemma between maintaining a given strategy with known return and exploring other strategies with unknown but potentially higher returns (Hills et al., 2015, see also, Komar et al., 2019). This aspect is present (as exemplified in the soccer example) but largely disregarded in the motor behavior literature.

#### Individuality, Equifinality, and Multifinality

A strong inference from SSA is that general principles of learning will be derived through observation of individuals interacting with the task rather than emphasizing the traditions of nomothetic *group* principles (Adams, 1987, see Molenaar, 2004). In our studies, we have found a range of differences between individuals in their initial conditions (Pacheco et al., 2017), motion through the task-space (Pacheco and Newell, 2015; Pacheco et al., 2017), and exploitation of task redundancy (Pacheco and Newell, 2018a,c). These characteristics in search-related processes explained large differences in performance and learning. As these aspects are discussed further in the upcoming sections, we do not provide any empirical example here. The ecological theory of perception and action has motivated studies that highlight individual pathways in motor learning (e.g., Molenaar and Newell, 2010; Kostrubiec et al., 2012), perceptual learning (Withagen and van Wermeskerken, 2009; Rop and Withagen, 2014), and motor development (Thelen et al., 1993, 1996).

A consequence of the discussion on individuality in search is that individuals from the same starting point might diverge in practice finding different solutions for the same motor task: multifinality. Thus, the practice on the same task condition can lead to a variety of solutions, depending on the initial conditions, search patterns, and the allowed redundancy of the task. As highlighted in the section “Traditional Assumptions in Skill Acquisition: Group- and Task-Based Learning,” this degeneracy needs to be a feature encompassed by theories of learning.

The approach of Direct Learning (Jacobs and Michaels, 2007) is related to SSA given the common origins in the ecological theory of perception and action but it does not consider the movement characteristics of search or the many expressions shown here of individual variation in learning. An initial assumption in Direct Learning was that the space of search (information space in their terms) determines the search and, thus, can dismiss the large variability between-individuals observed in the initial conditions through the end of practice. The role of variability (Higueras-Herbada et al., 2019) has been amended to deal with situations where the information space cannot guide behavior (Pacheco et al., 2017) but the theory still does not accommodate divergent patterns of change behavior between individuals.

Given the discussion on differences between individuals, it maybe more interesting to try to identify situations that elicit the opposite, equifinality. Overly constraining tasks might elicit this, but a more interesting situation would be one that occurs as a result of long-term practice. This would occur provided that practice is long enough to allow individuals to find a given sweet spot in the task-space (e.g., a tolerant region, see the section “Convergence to Tolerant Regions”).

#### Inherent Variability and Task-Space Perception

Since the early nineties, there is discussion on the role of the inherent variability that is observed over time in behavior – the so-called “noise” (e.g., Newell and Corcos, 1993; Riley and Turvey, 2002; Vereijken, 2010). Some researchers accepted the notion that inherent variability could be just more than a problem that must be minimized (e.g., a mechanism probing the system’s stability – Kelso, 1995), some emphasized an exploratory role (e.g., Riccio, 1993) or means to avoid bad solutions (e.g., Schöllhorn et al., 2009), while others still see it as a problem (Harris and Wolpert, 1998).

From SSA, all these viewpoints can be tested directly. In Pacheco et al. (2017), individuals had to minimize an error score that encompassed speed and accuracy while they did not know the weighting of each *a priori*. In one situation, there was a high weighting for speed and almost none for accuracy. Those who started emphasizing speed, because of the high variability resultant of the speed–accuracy trade-off (Fitts, 1954; Schmidt et al., 1979), rapidly perceived the lack of relation between accuracy and score (effect of initial condition!). Those who started emphasizing accuracy, nevertheless, would not perceive the relation provided the *lack* of variation. From the latter, only those who increased speed to the point of increasing variability (again because of speed–accuracy trade-off) perceived the relevant dimension of the task and modified the behavior accordingly. Thus, *inherent variability allows perception of the task-space, especially when variability allows perception of a new dimension of the task-space*.

Nevertheless, this *finding does not occur when individuals must find other solutions within the same dimension*. In a search task (Pacheco et al., in preparation) in which there was a single solution and two non-optimal solutions (local-minima), variability did not predict individuals perceiving the task-space. Two aspects are worth highlighting on this study. First, is that, as observed in Pacheco et al. (2017), some individuals never leave the wrong solution even knowing that they are far from the best performance (or the goal of the task). Second, in regard to inherent variability, this study started with the assumption that both increased or decreased variability could allow task-space perception. From Withagen and van Wermeskerken (2009) and Rop and Withagen (2014), decreased variability would allow individuals to differentiate actual errors from errors that emerge from inherent variability. From Schöllhorn et al. (2009) and Riccio (1993), increased variability would allow perception of the topology of the task-space allowing one to escape from local-minima. We found, contrary to both views, that variability allows perception of the task-space in *some* situations (new dimensions) but not always [see also Chow et al. (2008) on the relation of variability and change between movement patterns].

*Inherent variability can also induce qualitative changes in behavior*. For instance, Schmidt (1991) argued that receiving feedback close to the goal could induce maladaptive corrections as individuals would try to correct errors emerging from inherent variability rather than *actual* errors. Wolpert et al. (1992) showed that this would not be an issue as individuals usually demonstrate an error-deadzone (i.e., a bandwidth around the target where no corrections are made, see also Blackwell and Newell, 1996). Note that in both cases, variability is assumed to be high enough to be problematic for corrections to occur in terms of error (beyond the simplifying assumption of signal plus noise, see Riley and Turvey, 2002). In Pacheco and Newell (2018b), individuals performed a search task in which they had to find the target by increasing throwing distance in the tablet task [see the section “Virtual Throwing Task (Pacheco and Newell, 2015)”]. One of the targets was located closer than a second one. If variability is determinant in when individuals can or cannot correct in terms of error, the modifications on how to change behavior would occur in the farther target as individuals would vary more with farther throwing distances but not in the closer target. This was exactly what was found. While in the closer target the relation between change and error was proportional (as in Figure 5D), the farther target showed a discontinuous relation with a change independent of error when they were approached the farther target (Figure 9B).

#### Emergence of Compensatory Mechanisms

Considering the observed exploitation of body/task redundancy, several approaches have been developed to quantify such exploitation. One of them is the Uncontrolled Manifold (Scholz and Schöner, 1999; Latash et al., 2002). The main aspect posited in the UCM approach is that the control of a given variable will result in a large variation along the uncontrolled manifold (*v*UCM, redundant space) – maintenance of the relevant variable in the desired state – while the variation in the dimension that represents non-desired changes in the controlled variable will be minimized (the orthogonal space – *v*ORT). In learning, one would expect that this would result in the performance variable representing, at some point in practice, the controlled variable. This would lead to the elemental variables maintaining the performance variable stabilized (large *v*UCM and small *v*ORT).

The tenets of UCM (Martin et al., 2009, 2019) assume that the structure arises from the decoupling of ORT and UCM spaces by the central nervous system, back-coupling mechanisms, and noise at neuronal and muscular levels. These developments emphasize mechanisms of an already proficient individual in the task. Here we want to pursue an explanation that can explain how this emerges in search.

**FIGURE 8:**
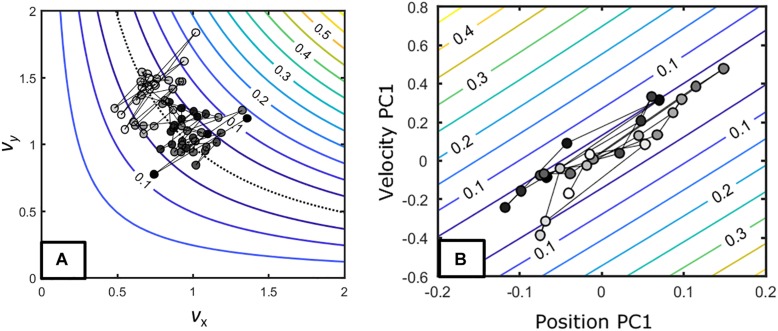
**(A)** Task-space and trials of an exemplary individual in Pacheco and Newell (2015). **(B)** Task-space and trials of an exemplary individual in Pacheco and Newell (2018c). These figures exemplify how the task constraints might influence how individuals achieve a UCM-like distribution (see the text).

A first consideration is that the UCM structure emerges differently depending on the task constraints. This is exemplified in Figure 8 that shows the same tasks as in the sections “Virtual Throwing Task (Pacheco and Newell, 2015)” and “Throwing Task (Pacheco and Newell, 2018c).” Although one could state that in both cases *v*UCM would be higher than *v*ORT we clearly see that in Figure 8A the trial-to-trial variation occurs orthogonal to the goal-space while in Figure 8B it occurs along the goal-space.

These differences emerge from the way the task constrains behavior. In the former task, the release parameters are defined by the peak velocity of the movement, while in the latter, the individual can choose when the release occurs in terms of the velocity profile. Recent experiments (Cohen and Sternad, 2012; Nasu et al., 2014; Zhang et al., 2018) have demonstrated that when individuals can choose when to release, they adjust their movement to occur along the goal-space. This makes the moment of release parameter to not matter and, as we observe in Figure 8B, to result in trial-to-trial variation along the goal-space. This does not emerge in Figure 8A because the task constraints do not afford such a strategy. Thus, *although in both cases UCM-like structure emerged (large variation along the goal-space and small variation on the orthogonal direction), these structures emerged from different constraints*: one emerges from stabilization at the trial-to-trial dynamics of the ORT with its *v*UCM resulting from drift in coordination while the other emerges from trajectory along the goal-space with no strong trial-to-trial dynamics in the ORT.

A second consideration is whether this compensatory mechanism *always* emerges – being a principle of learning. A firm position is impossible, but we do not anticipate this to be the case. Although the UCM literature in a range of tasks and contexts support this idea (e.g., Papi et al., 2015; Mangalam et al., 2018; Vaz et al., 2019), the UCM-like distribution of the data seems to occur when there is non-desired variability to be compensated. For instance, Latash (2010) has provided examples that in the middle stage of learning the UCM-like distribution might be more pronounced than when individuals are more proficient. Also, special populations demonstrate higher or equal UCM-like distribution than their healthy (or younger) peers (Black et al., 2007; Golenia et al., 2018; Greve et al., 2019). Furthermore, many of our experiments when analyzed for each individual do not show increased UCM and decreased ORT as would be expected (non-published results).

#### Convergence to Tolerant Regions

Another approach developed to study the redundant many-to-one relation between movement parameters and performance is the Tolerance, Noise, and Covariation (TNC) decomposition (Müller and Sternad, 2004; Cohen and Sternad, 2009). In this approach, the experimenter can quantify how much aspects of variability *cost* to performance during learning. The TNC approach essentially suggests that the search process is driven by exploration for, and exploitation of, regions of the task-space where inherent noise of the system “matters less” (Sternad, 2018). This is captured by the term of “tolerance” within the TNC framework.

The core task employed in this approach is the (virtual) skittles pub-game where individuals throw a ball hanging in a pole to hit a target. This task is of relevance given that, depending on the target placement, there is a more tolerant region of the task-space. In this task, individuals usually converge to this region as they practice. Sternad (2018) has discussed the convergence to the most tolerant region as a general phenomenon in learning that we now consider in the context of searching.

First, *not all tasks have differential tolerance around the goal-space*. We can consider for instance the bimanual isometric force task presented in Figure 7. In contrast to the skittles pub game this task provides a linear goal-space. As a result, anywhere along this line maintains the same tolerance to force fluctuations. Thus, convergence to the most tolerant region of the task-space would occur *if* the task has differential tolerance. Second, *even when individuals practiced for a long period (as in Cohen and Sternad, 2009), the experts did not converge to the same region of the space* – some found more tolerant regions than others. Third, *the TNC approach does not account for how such convergence to more tolerant regions would occur in search behavior*.

We, nevertheless, see tolerance as an interesting aspect that can shape how individuals perceive and change during search. Considering the bimanual isometric force tracking task, for instance, how individuals perceive the task-space would be dependent on the interaction of informational and organismic constraints (e.g., force variability, tendencies in action). For instance, increased fatigue in a given finger would lead to increased variability in force production. This could elicit changes in how much each finger contributes in the average (see Singh et al., 2012) demonstrating a change on *where* individuals stay in the task-space given modifications on organismic constraints.

Sternad et al. (2011) sought to differentiate the emphasis on tolerance or inherent variability using the skittles task. In a situation which the goal-space was defined in terms of a single velocity, exploiting lower velocities would reduce velocity-dependent variability, but the most error tolerant region were to be found with increasing velocity. Although it was claimed the results supported the latter, convergence to a common velocity was not observed and there was not the expected velocity-increasing-variability relation.

A factor not considered is that individuals who preferred higher velocities may be searching another elemental variable. In the section “Task-Space and Goal-Space,” we highlighted that one can change the task coordinates to better understand strategies (e.g., Figures 4B,F). In the case discussed, increase in velocity leads to a reduction in timing variability (Newell et al., 1979) and individuals could be exploiting such relation. Provided the unique case where velocity is free to vary, the timing of release would determine the success of each throw (see also Cohen and Sternad, 2012; Zhang et al., 2018). This could have modified the expected increased-velocity-increased-variability relation (see Pacheco et al., 2019).

One might consider that *inherent variability would afford perception of tolerance in the task-space*. Nevertheless, as highlighted earlier, it is not explicit how variability should relate to perception of tolerant regions – or any other properties of task-space. It is interesting to note that, within the TNC framework, there is an implicit link between noise-cost and tolerance, in that if noise is too great certain regions would be rendered intolerant. The implication is that individuals would find certain regions tolerant while others would not. Thus, an understanding of the relation between intrinsic variability and tolerance in regions of the task-space might explain the divergence in practice.

### Learning Curves

The field of motor learning has followed the traditions of psychology and learning theory in the analysis of learning curves (Bower and Hilgard, 1981; Newell and Rosenbloom, 1981; Newell et al., 2006) or, as some prefer to label them, performance curves (Schmidt and Lee, 2005). Although theoretical claims were derived from such curves (e.g., Fitts, 1964; Newell and Rosenbloom, 1981; Taylor and Ivry, 2012), two issues appear. First, the laboratory tasks used have tended to be those where participants can already produce the task-relevant coordination mode leaving the function of change to be about parameterization of a learned coordination pattern (Newell, 1985). Second, the one-to-many relation between performance and movement patterns creates the issue of interpretation of the learning curves. In other words, if a given pattern of performance change can reflect different types of change in movement patterns, how can we interpret the given performance change uniquely (e.g., it relates to a unique “learning stage”)? In considering learning as searching, we can show that the different types of learning curves discussed in the literature (i.e., power-law, exponential, and sigmoidal) emerge from the interaction between individual and task-space. Continuities and discontinuities can imply a number of processes in the individual pathways of change (Molenaar and Newell, 2010).

An instructive example is the roller-ball experiment employed by Liu et al. (2006, 2010). This task paradigm requires the individual to maintain an inner ball to keep rotating through movements in an outer ball. Considering the performance in terms of success and failure, there is a variable (imposed acceleration) that individuals must learn to control to achieve success. Although individuals might continuously change this variable, given the relation between this variable and performance (task-space), there will be, inevitably, a sigmoidal performance outcome curve. If the task-space was the only reason for the emergence of the curve, when considering the average acceleration of the ball within a trial as performance measure (Liu and Newell, 2015) the sigmoidal curve should not be observed. For some individuals, this occurred: the change was better represented by a gradual change. Nevertheless, other individuals still presented the sigmoidal curve, implying some other type of discontinuity in the learning process. One could speculate different strategies (see Pacheco et al., 2017) or coordination patterns employed.

Thus, we propose that one can only infer from performance curves if accompanied with properties of the search process employed. The performance outcome score reflects the end product of the search process for the task-relevant solution. The analysis of learning curves, therefore, represents the macro level analysis of the product of the confluence of processes of learning. In this capacity, the performance outcome measure alone does not directly reflect the search strategy and the processes of learning. The performance score needs to be supplemented with other levels of analysis such as those shown here in the experimental examples of searching as learning.

### Learning Factors Through SSA

In the following section we discuss traditional factors of learning (e.g., task instructions, practice conditions, etc.) through an SSA lens. Instead of determining a single role for each learning factor identified in the literature, we discuss them in terms of how and when they constrain the search space and/or modify the motion through the space (see also Newell, 1991, 1996).

#### Factors Constraining the Search Space

A primary aspect for theory and practice is how to manipulate aspects of the task to guide learners to specific aspects of the to-be-learned task facilitating skill acquisition. There are plenty of learning strategies discussed in the literature. For instance, learners can be told to perform a given movement pattern in a specific way (e.g., instructions), mirror an idealized model of the coordination pattern presented visually (e.g., observational learning), or simply discover an appropriate way of performing for themselves (e.g., discovery learning).

Newell and Ranganathan (2010) conceptualized augmented information (such as instructions, demonstrations, and cueing) as providing forward-looking information directing the learner toward relevant aspects of the to-be-learned skill. In this way, augmented information can be thought of as an informational constraint on action. In our terms, this constraint would limit the search space of the task, guiding the learner to the most appropriate space of the task to perform. In some cases, this might be over-constraining. Newell and Ranganathan (2010) suggested that particular movement forms should not be imposed; the teacher should use instruction as a means of facilitating the search for a coordinative solution. Some authors go further in arguing that the absence of specific verbal instruction (discovery learning) would be superior to when instructional demands are given. Discovery learning would provide opportunity for individuals to explore the perceptual motor-workspace to a greater extent (Green and Flowers, 1991; Hodges and Lee, 1999; Hodges and Franks, 2001).

Clearly, the effectivity of discovery learning depends on the task. Several studies have shown that discovery learning in more complex tasks takes longer to find and stabilize a coordination solution – resulting in worse performance overall (Janelle et al., 2003). Additionally, some degree of information constraint is always present as soon as the goal of the task is made clear.

Demonstrations clearly prioritize a given movement pattern and, even though the learner might not exactly reproduce the observed movement, the learner will perform the task in a search space “around” or intending to get to the demonstrated pattern (see Al-Abood et al., 2001). In the same way, cueing direct the learners’ attention to a relevant aspect of the task constrain the learner to perform *based* on this aspect. One could question whether such aspect of the task being cued would inevitably emerge in movement as search is allowed to occur without interference.

The area searched in the task-space and perceptual-motor workspace might be modified by the task constraints themselves. Hristovski et al. (2011) presented the summary of findings indicating that, for instance, in boxing a heavy-bag, the distance of the athlete to the bag modified the range of behaviors observed. Their modeling demonstrates how different levels of behavior – from types of punches (movement patterns), to side of punching, to angle of punching within a given movement pattern – emerged as the distance parameter was modified. It would be an interesting avenue of research to understand the relation between changes in level of search in constant task constraints (the section “Perception and Action Search Within a Single Level”) versus changes in levels of search in variable task constraints.

#### Factors Modifying the Motion Through the Space

Beyond constraining, factors of learning might alter how motion through the space will occur. One way is by altering the information that individuals receive about their performance. Here, we will consider mainly knowledge of results (KRs) but the discussion is applicable to different types of information manipulation [e.g., concurrent feedback, knowledge of performance (KP), etc.].

Knowledge of result has historically been viewed as the most effective information to provide the learner of a perceptual motor skill (Annett, 1969; Adams, 1971). The view considered KR to be *sufficient* to strengthen a task relevant reference trace (Adams, 1971) or a schema (Schmidt, 1975), and also intrinsic corrective mechanisms (Salmoni et al., 1984). Much of this interpretation came from single degree of freedom positioning tasks (and the like) that dominated the study of motor control and learning for a period of time. It is questionable, however, whether this role for KR actually holds when considering the whole spectrum of tasks, levels of learning, etc.

In single dimension tasks, KR maps to the single degree of freedom of the positioning task that is to be controlled. Note, however, that if we consider other single dimensions tasks, more than a single trial must be performed to allow individuals to map variations within a given coordination to change in KR when the relation is not explicit. For tasks with more dimensions, KR becomes just a statement of what was done but not what should be done next (Fowler and Turvey, 1978). The question becomes in which direction of the higher-dimensional space of joints, muscles, etc. one should move when KR is, for instance, 5? Can we update reference, schema, and correction mechanisms? The argument holds for other types of information.

Many studies, however, demonstrate the effectiveness of KR in learning. The ecological theory for perception and action addresses the issue. First, individuals are constrained by the coordination patterns that emerge from the interaction between task, organism, and environment (Newell, 1986). Thus, lower-dimensional spaces are to be explored, not the full space of elemental variables. Second, Gel’fand and Tsetlin (1962) and Fowler and Turvey (1978) pointed out that individuals must be able to perceive the properties of the task-space in order to find solutions. This could only occur if, beyond the usage of KR, individuals would attend to higher dimensional variables (e.g., change in KR – ΔKR, relative change in KR – ΔKR/KR, and so on). Thus, the learner must have information for change in the movement pattern (see Newell et al., 1985; Kernodle and Carlton, 1992).

**FIGURE 9:**
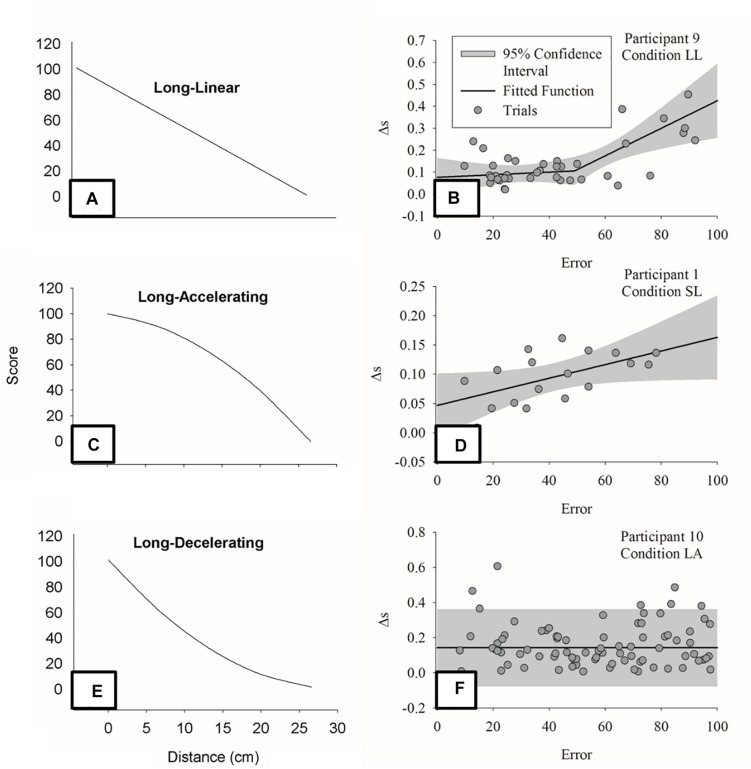
Panels **(A,C,E)** show three task conditions from Pacheco and Newell (2018b). These differ in terms of the relation between error and distance to the target while the target distance itself was constant. Panels **(B,D,F)** show three exemplary data sets that cover the three types of relation between error and change: **(B)** discontinuous, **(D)** proportional, and **(F)** constant.

Note that in altering the type of feedback, one constrains the learner to find the means to change in accordance to such type of information. The section “Bimanual Isometric Force Task (Lafe et al., 2016)” showed, for instance, that manipulation of intermittency in concurrent feedback induces large shifts in the coordination between hands as higher frequency of presentation seems to be required for compensation between hands to occur (Lafe et al., 2016, see also Withagen and Michaels, 2005). Another paper helps to elucidate the emergent nature of how KR interacts with action. Pacheco and Newell (2018b), discussed in the section “Perception and Action Search Within a Single Level,” included two more target distances that only modified the relation between KR and distance, as shown in Figures 9A,C,E. Even though the task was the same, individuals demonstrated different functions relating KR and change in behavior (i.e., linear, piecewise, and constant) depending on the task-space performed. Figures 9B,D,F show these different trends. That is, there is not a fixed relation between KR and behavior: it emerges from action and perception on the task-space.

Finally, the factors outlined in the previous section that decrease the available space of search might also alter how individuals search within the space available. By constraining the search space, demonstrations, focus of attention, and other factors alter the initial perception-action coupling employed in a task. As discussed earlier, the way individuals act alters the informational variables they can attend and perception, in turn, delimits the afforded actions. Thus, in changing the initial perception and action coupling employed, these learning factors alter how the ongoing perception-action coupling changes; they alter how search occur. That is, the capacity of acting, which is a function of experience, alters the affordances perceived, which in turn alters the experience in practice.

We have already pointed to some evidence on the importance of initial conditions (Pacheco et al., 2017, see also the section “Individuality, Equifinality, and Multifinality”) but Liu et al. (2006, 2010) directly manipulated them. Using the roller-ball paradigm (the section “Learning Curves”), they manipulated the initial speed of the inner ball modulating the initial region of the task-space in practice. They found that, indeed, by altering the initial conditions of the learner, one could predict those who would succeed in the task.

### Exploration of the Task-Space and Transfer

So far, we have discussed many aspects that refer to how search allows one to understand change during practice. Nevertheless, if search refers to learning, more than explaining regions explored is necessary. In this section, we briefly refer to our results on transfer as an indication of how search modifies the perceptual-motor workspace.

Our studies have demonstrated that the differences in the end-state of practice – given their different initial conditions and search patterns employed during practice – do predict performance in transfer tests. Two experiments employing the throwing paradigm (Pacheco and Newell, 2018a,c) showed that the learned movement patterns during learning could explain, more than the practiced conditions of practice (variable or constant), the performance in transfer. This supports our working hypothesis in the introduction that searching might be as or more important in learning than the task conditions. An interesting question, then, is how to manipulate task conditions (beyond the current approaches, e.g., Ranganathan and Newell, 2013) to induce the learner to find and learn generalizable solutions.

More interesting, however, is the fact that the patterns of search must be considered – even when the same end-state is observed. Hodges and Lee (1999), for instance, showed that discovery-learning and instructed groups in a bimanual task converged to the same performance during practice. Nevertheless, the discovery-learning group demonstrated better transfer results which was considered as evidence to support enhanced exploration.

Pacheco and Newell (2015) measured different patterns of exploration using a range of dependent variables (from dispersion to trial-to-trial structure) to assess the effect of search on transfer. The task used was the virtual throwing in the tablet [the section “Virtual Throwing Task (Pacheco and Newell, 2015)]. The results showed that, first, only trial-to-trial measures of exploration predicted transfer and, second, that maintaining a balanced strategy between exploring and exploiting was the most successful.

The fact that the search patterns *per se* predict the ability to generalize to new situations (in some cases independent of the experimentally imposed practice condition) is intriguing. We speculate that there are some informational variables that are picked up only when given search patterns are employed, allowing individuals to generalize to regions of the task-space never visited. Note that this is/was not dependent on the overall area visited but of the trial-to-trial pattern of search (Pacheco and Newell, 2015). The perception of these informational variables might facilitate what Harlow (1949) described as learning-to-learn: a capacity to generalize to situations that go beyond the current one experienced.

## Expanding Change as Searching

In the above sections we provided interpretation of existing positions on learning and speculations as to the role of certain factors in constraining search behavior, as well as how search modifies the workspace. Here we elaborate further on the implications for adjacent areas of skill acquisition if they were to adopt the SSA initiative.

### Sports Training and Rehabilitation

It is useful to extend a bridge that shows that our theoretical approach on SSA is applicable to many other domains. It could be said that current sports training and rehabilitation approaches fall victim to the same concerns presented in the section “Traditional Assumptions in Skill Acquisition: Group- and Task-Based Learning.” Individuality is regularly discussed and acknowledged among trainers, yet there has not been significant advances to understand the individual nature of change occurring within these intervention programs (Bialosky et al., 2017). The SSA is well positioned in this case to provide therapists and trainers a deeper understanding of the change occurring with their clients and/or athletes.

The parsimonious position holds that principles of rehabilitation, or re-learning are the same as those of learning and development (Newell and Verhoeven, 2017). How the perceptual-motor workspace is constrained by strategies of learning should not be considered unique to healthy normal populations – it can be extended to whole skill continuum (rehabilitation to high performance). Learning occurs as the search for and change of equilibrium regions within the workspace that also satisfy the task demands. Injury or development of a movement disorder can be seen as additional constraints on action possibilities, modifying the search. What remains to be investigated is how specific trauma (e.g., ACL tear, stroke, etc.) would modify the search and search space.

Just as pathology could constrain search, therapy could release constraints. Common manual therapy techniques (e.g., myofascial release) have even been suggested to directly affect both peripheral and spinal mechanisms to improve – for example – joint range of motion and manage recovery and pain (Beardsley and Skarabot, 2015; Kalichman and David, 2017; Monteiro et al., 2017). Thus, it stands to reason that therapists may modulate the properties of the perceptual-motor workspace of the patient, potentially through regulation of muscular tone (Cacciatore et al., 2011a, b; Profeta and Turvey, 2018). Nevertheless, the exact mechanisms of manual therapy are not well established (see Bialosky et al., 2009, 2017).

Recently, the efficacy of contemporary models of training and rehabilitation has been questioned (Stergiou et al., 2006; Schöllhorn et al., 2009; Vaz et al., 2017; Silva et al., 2018). Silva et al. (2018) demonstrate, in comparison to models based on information processing, the utility of ecologically grounded methods of rehabilitation. Also, the role of variability and exploration has been advocated in sports practice (e.g., Orth et al., 2018a, b). These recent advances would be greatly supported by the consideration on the nature of search strategies.

Finally, all interventions can be thought of as a form of practice to induce transfer. Each exercise is done for the express purpose of generalizing the skill learned in one situation to many (e.g., seated leg extensions to locomotion). As we have seen, understanding the search patterns during practice is highly relevant to predict transfer (the section “Exploration of the Task-Space and Transfer”) and, in this case, the effectiveness of a training or a rehab program.

### Motor Development

As the usage of the term skill acquisition implies, the theoretical and operational aspects presented in earlier sections are all applicable to the study of motor development and, more generally, the construct of change. In this section, we briefly outline a few representative issues that could be fruitfully addressed in motor development with the search strategy approach.

A long-standing question in motor development is whether there is an order to which the individual component variables (joint motions) are frozen or released either individually or in combination (coupled) as a function of development. Gesell (1929) proposed that the developmental order to the effective functioning of the joint space motions (DFs) was anatomically organized in terms of realizing an unfolding directional control: namely, cephalo-caudal, proximal-distal, and ulnar-radial. A competing account holds that is the neural development of the central and peripheral nervous systems that drives the order of engagement of the functioning DFs (McGraw, 1945). Another hypothesis is that the segment masses of the limbs and torso in action are the primary determiners of the unfolding order to children’ coordination and control in movement skills (Thelen and Smith, 1994). The systematic manipulation and analysis of the relevant variables, including the task demands (Newell, 1986), could help unravel this still open and basic question on the emergence of the fundamental motor skills (e.g., sitting, standing, reaching). An analysis of the search strategy used to realize performance change in the task-space as a function of the particular motor skill would be foundational finding from a developmental perspective.

An interesting possibility is to see the classic freezing-and-freeing stages of Bernstein (1967) as a process that facilitates search over development. He proposed that, initially, the system would avoid dealing with the complexity of the body by decreasing the number of degrees of freedom to a minimum. In search terms, this would mean that the whole space of possibilities has been decreased to a minimum of dimensions, allowing exploration of the task-space to occur through parameterization only. As relevant informational variables of the task-space had been attended, and as organismic constraints and task goal requires (see Newell and Vaillancourt, 2001), new dimensions are explored, degrees of freedom are freed, and, later, reactive phenomena are exploited. From our results, we can speculate that inherent and external variability allow perception of the most appropriate dimensions to be explored and how to exploit reactive phenomena (Schöllhorn et al., 2009).

Brakke and Pacheco (2019) have already considered search in the motor development domain. In following the lead of Thelen et al. (1993) that detailed how infants explore parameters of movement to facilitate the emergence of reaching, Brakke and Pacheco (2019) mapped the many possible parameters that toddlers could search on to stabilize the performance of the anti-phase pattern in drumming. As in all search studies, they found individual strategies based on different movement parameters (i.e., frequency of oscillation, amplitude ratio between limbs, etc.) that allowed toddlers to break the tendency to perform the in-phase pattern and perform the required pattern.

The fact that one can apply SSA in the motor development domain is not surprising as many authors have highlighted the huge influence of exploratory patterns in development (e.g., Eppler et al., 1996). SSA, differently, is just investigating how these emerge in terms of systematic time-dependent patterns and what are the effects of different exploratory patterns in development. The importance of this study goes beyond showing that SSA can nicely demonstrate how individuals explore the dynamics described in the HKB (and its developments, see de Poel et al., 2009) to acquire a new skill – an example of the integration discussed in the section “Introduction.”

## Closing Comments

In this paper we have drawn on the assumption of learning as searching to examine analytically and experimentally the multiple processes of change that emerge in perceptual-motor skills through practice. Our approach to search strategies continues to build within tenets of the ecological theory of perception and action (Turvey, 1992; Warren, 2006; Profeta and Turvey, 2018) and in particular the original framework of search processes and the perceptual-motor workspace (Kugler and Turvey, 1987; Newell et al., 1989). A distinctive emphasis here, however, is on individual analysis of the change in performance and its expressions in the different behavioral frames of reference (Saltzman and Kelso, 1987; Newell and Liu, 2014): namely, goal, task, perceptual-motor workspace, and individual component DFs.

We have provided evidence for the central proposition on the nature of search strategies locating and creating stable equilibrium regions in the perceptual-motor workspace and how these strategies relate to the emergent movement forms in the acquisition of coordination, control, and skill. In this regard we have brought together the role of intrinsic dynamics (Kelso, 1995) and the emergence of processes of search in task space that can be independent of the gradient descent properties of the landscape of both task and intrinsic dynamics (Newell et al., 1989). This possibility opens the question on the processes of perception of informational variables that grasp macroscopic properties from task and perceptual-motor workspace that leads to divergent search patterns (e.g., Jacobs et al., 2011; Michaels et al., 2017). It also opens the question as to the learner’s priorities for using information on one level or another in the search process for learning.

In both discrete and continuous perceptual-motor skills we have shown that a search strategy analysis reveals new aspects about change and learning beyond those from the traditions of task-based assumptions and averaged performance outcome over time. Through the search process, individuals adapt to the pick-up of task relevant perceptual variables and change their movement form according to the stability of the performed action and its outcome in relation to the task demands. Contemporary experimental findings have revealed some clues as to how the search process occurs given the interaction of individual intrinsic dynamics in the context of task requirements by extracting the commonalities arising from the individual differences in practice. Also, we elucidated and evaluated potential principles that drive the change – e.g., exploitation of more tolerant task-space solutions, emergence of compensatory mechanisms, and inherent variability. One might recognize that the potential benefits of degeneracy of the system for the learner become the potential challenge for the researcher.

Examining search behavior at goal, task, and perceptual-motor workspace adds to this challenge of seeking order in the time-dependent processes across variables – the search behavior. All these challenges and questions can be approached from the contemporary developments in search processes for the learning of perceptual motor skills.

## Data Availability

The raw data supporting the conclusions of this manuscript will be made available by the authors, without undue reservation, to any qualified researcher.

## Author Contributions

MP, CL, and KN derived the idea, and wrote and revised the manuscript.

## Conflict of Interest Statement

The authors declare that the research was conducted in the absence of any commercial or financial relationships that could be construed as a potential conflict of interest.

## Footnotes

1 Because of tradition, nevertheless, we will still call it as “skill acquisition.”

2 This can be said to have roots in Bernstein’s ideas: “The process of practice toward the achievement of new motor habits essentially consists in the gradual success of a search for optimal motor solutions to the appropriate problems” (Bernstein, 1967, p. 362).

3 As noted by a reviewer, attunement (and even action changes) might occur without over action. Wilson et al. (2010), for instance, have shown that individuals learned to perform a new relative-phase in bimanual coordination observing others performing without physical practice.

4 Although we refer to *spaces*, the best term probably is manifold. Spaces usually refer to geometric spaces such as the Euclidean space. Manifolds are topological spaces that locally resemble Euclidean space near each point (Abraham and Shaw, 1992).

5 Note that there is no necessity for plotting the data. It is, nevertheless, always recommended to understand the nature of the data – even if for few participants – before drawing firm conclusions.
